# Rate of complications in scoliosis surgery – a systematic review of the Pub Med literature

**DOI:** 10.1186/1748-7161-3-9

**Published:** 2008-08-05

**Authors:** Hans-Rudolf Weiss, Deborah Goodall

**Affiliations:** 1 Asklepios Katharina Schroth Spinal Deformities Rehabilitation Centre, Korczakstr. 2, D-55566, Bad Sobernheim, Germany; 2 163 Sandringham Road, WD24 7bh Watford, London, UK

## Abstract

**Background:**

Spinal fusion surgery is currently recommended when curve magnitude exceeds 40–45 degrees. Early attempts at spinal fusion surgery which were aimed to leave the patients with a mild residual deformity, failed to meet such expectations. These aims have since been revised to the more modest goals of preventing progression, restoring 'acceptability' of the clinical deformity and reducing curvature.

In view of the fact that there is no evidence that health related signs and symptoms of scoliosis can be altered by spinal fusion in the long-term, a clear medical indication for this treatment cannot be derived. Knowledge concerning the rate of complications of scoliosis surgery may enable us to establish a cost/benefit relation of this intervention and to improve the standard of the information and advice given to patients. It is also hoped that this study will help to answer questions in relation to the limiting choice between the risks of surgery and the *"wait and see – observation only until surgery might be recommended"*, strategy widely used. The purpose of this review is to present the actual data available on the rate of complications in scoliosis surgery.

**Materials and methods:**

Search strategy for identification of studies; Pub Med and the SOSORT scoliosis library, limited to English language and bibliographies of all reviewed articles. The search strategy included the terms; 'scoliosis'; 'rate of complications'; 'spine surgery'; 'scoliosis surgery'; 'spondylodesis'; 'spinal instrumentation' and 'spine fusion'.

**Results:**

The electronic search carried out on the 1^st ^February 2008 with the key words "scoliosis", "surgery", "complications" revealed 2590 titles, which not necessarily attributed to our quest for the term "rate of complications". 287 titles were found when the term "rate of complications" was used as a key word. Rates of complication varied between 0 and 89% depending on the aetiology of the entity investigated. Long-term rates of complications have not yet been reported upon.

**Conclusion:**

Scoliosis surgery has a varying but high rate of complications. A medical indication for this treatment cannot be established in view of the lack of evidence. The rate of complications may even be higher than reported. Long-term risks of scoliosis surgery have not yet been reported upon in research. Mandatory reporting for all spinal implants in a standardized way using a spreadsheet list of all recognised complications to reveal a 2-year, 5-year, 10-year and 20-year rate of complications should be established. Trials with untreated control groups in the field of scoliosis raise ethical issues, as the control group could be exposed to the risks of undergoing such surgery.

## Background

Scoliosis, as a general medical term is better known as a lateral curvature of the spine [1], and is conventionally measured using the Cobb angle technique of X-rays taken of the coronal plane view [2]. But as it presents clinically, the condition is actually a much more complex deformity and to correctly measure and define the different effects it has upon the human spine it is necessary to use 3D terminology along with observations taken on the three anatomical planes [1].

**Figure 1:**
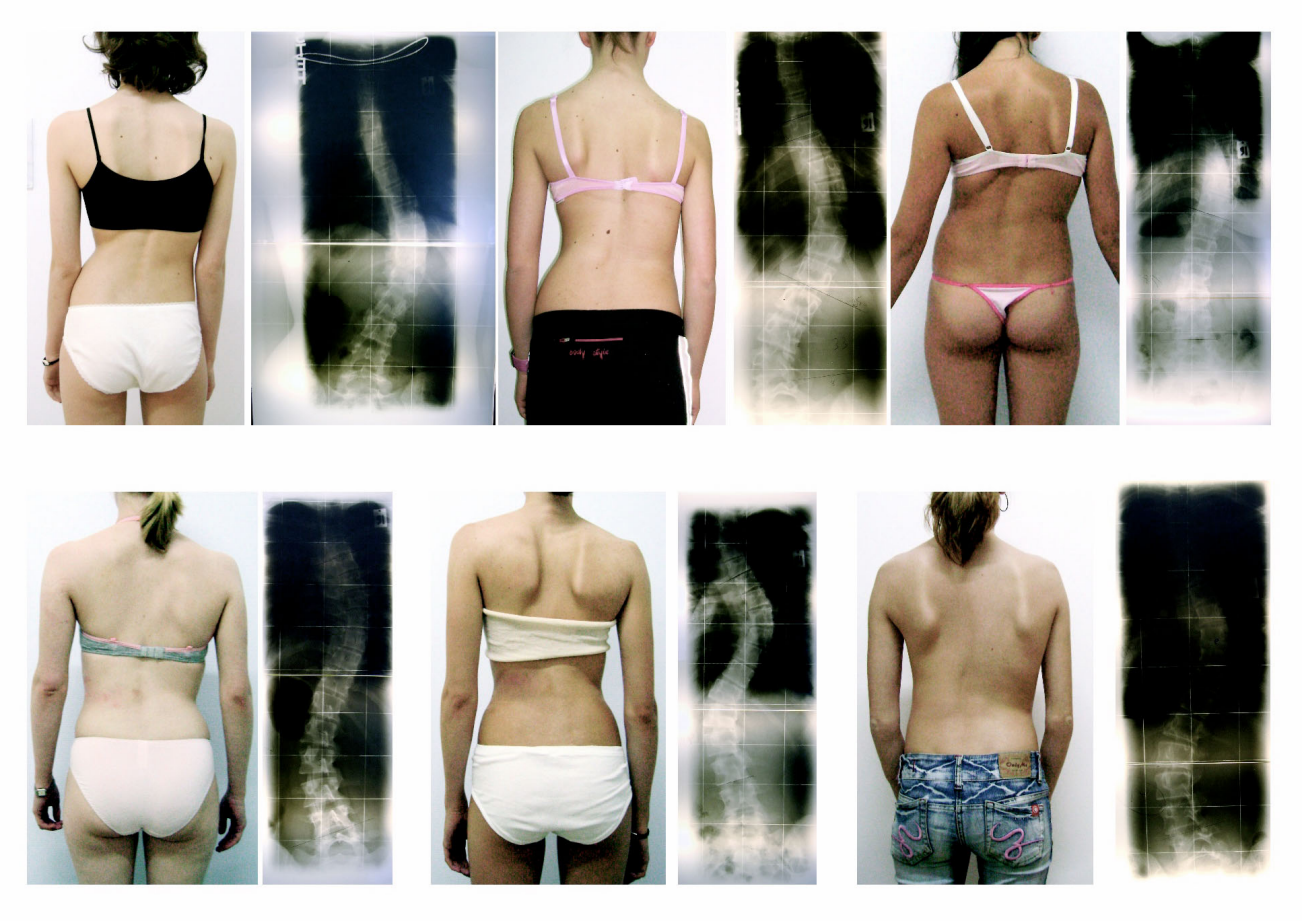
**Similar Cobb angles clinically may look different depending on curve pattern**. All patients on this figure have a Cobb angle of 40 degrees. As can be seen, the more decompensated a curve, the more visible the deformation. Double major curvatures are compensated; the most stable curves present after the end of growth [4] and therefore rarely requiring surgical treatment.

The underlying cause of scoliosis may on some occasions be clearly determined, such as congenital changes, or neuropathic or myopathic conditions, or a form of degenerative spondylosis. In the majority of cases, the causes are unfortunately unknown and have come to be known as having 'idiopathic' scoliosis [3]. Adolescent Idiopathic Scoliosis (AIS), the most common form of scoliosis, is a three-dimensional structural deformity of the spine and of the trunk, occurring in otherwise healthy children during puberty, while early onset idiopathic scoliosis occurs before puberty [4]. AIS has been classified according to specific curve patterns and these patterns clinically may appear more or less pronounced (Figure 1).

Historically, in central Europe the treatment for AIS – and for some other forms of scoliosis as well – includes; Physiotherapy (PT) on an outpatient basis; Scoliosis In-patient Rehabilitation (SIR); corrective bracing and surgery, with or without spinal fusion [5-7]. Conservative Scoliosis Management is usually regarded as effective when curvature progression has been stopped below specific limits, although parameters other than curve progression may play an important role in terms of outcome [4-6].

**Figure 2:**
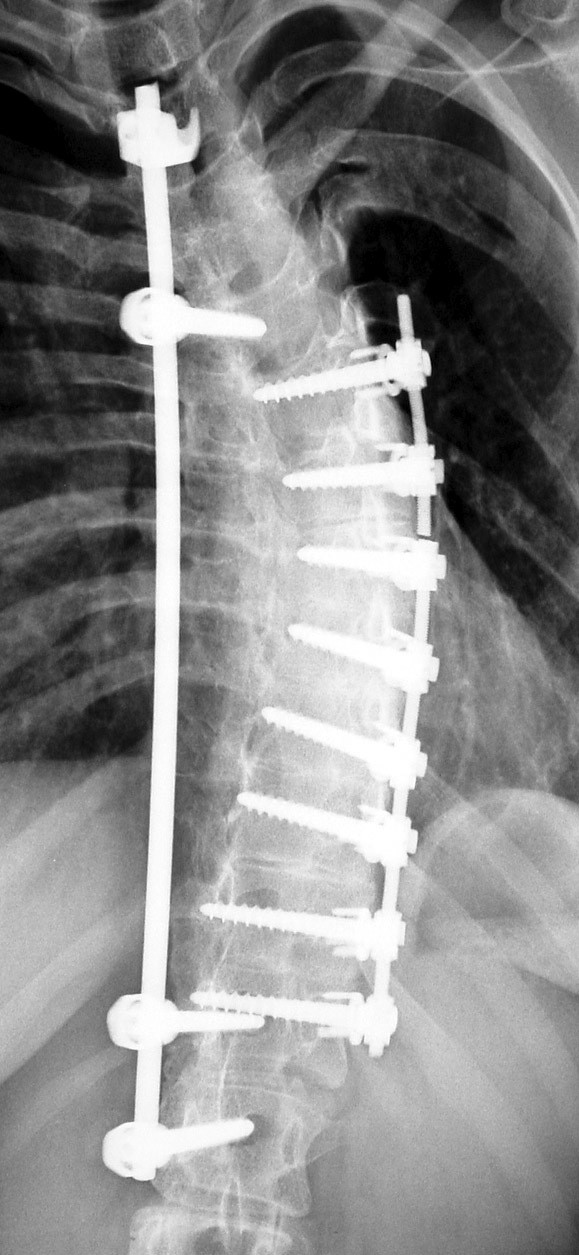
**Failure of spinal fusion often requires salvage surgery**. Failure of the ventral instrumentation (VDS): An additional dorsal rod was implanted to stabilize the spine.

Spinal fusion surgery, which is recommended when magnitude of curvature exceeds 40–45 degrees, has been used as a treatment for nearly a century [8-10]. The aims and goals of surgery have varied widely. Early hopes that spinal fusion could be used to leave the patient with a mild residual deformity were not successful as a third of patients lost all postoperative correction within 1–10 years post surgery [11]. Expectations have been revised to the more modest goals of preventing progression, restoring 'acceptability,' and reducing curvature. In spinal fusion the vertebrae are accessed by posterior, anterior, or thoracoscopic incision. The main principle these surgical techniques have in common, is the use of the spine as a structural scaffold, cementing the parts onto this via a bone paste, giving it an overall straighter shape. [10,12-14]. These surgical methods are based on the expectation that this operation will heal well and remain sturdy for the lifespan of the patient. Steel rods, screws, wires etc. have been used to reinforce the stability of the spinal fusion [13-18] and the choice of the instrumentation used is based upon the preferences of the surgeon [19-23]. The specific choice of these has been reviewed recently [21-23]. Failure of spinal fusion requires re-operation to restore curvature correction [19] (Figure 2). In a recent review [24], the different kinds of complications which may arise during or after scoliosis surgery have been listed:

### Complications of Spine Surgery independent of aetiology

In principle, all kinds of complications may occur in all scoliosis aetiologies [24]. However, in the otherwise healthy subjects with AIS the incidence of major complications may not be as high as in neuromuscular disorders [24]. Before outlining the incidence and prevalence of complications (Tables 1, 2 and 3) it is of primary importance to describe the possible complications independent of the aetiology.

**Table 1:** Pooled rate [122] of complications for the different aetiologies

	Studies	Average rate	Range
Neuromuscular Scoliosis	22	35% (SD 21)	0 – 89%
Adult Scoliosis	11	44% (SD 24)	10 – 78%
Idiopathic Scoliosis	11	20% (SD 22)	0 – 73%*
Early onset Scoliosis	1	48%	
Congenital Scoliosis	4	14% (SD 23)	0 – 48%
Congenital Heart Disease	1	27%	

This table shows the wide range of variability of the overall complication rate in the different entities. * In the IS group one study on fusion down to the pelvis [69] showed the highest rate of 73%.

**Table 2:** Pooled rate [122] of major complications for the different aetiologies

	Studies	Average rate	Range
Neuromuscular Scoliosis	17	17,4%	0 – 39%
Adult Scoliosis	6	30%	10 – 62%
Idiopathic Scoliosis	7	8,6%	0 – 37%*
Congenital Scoliosis	3	3%	0 – 9%

The pooled data [122] seems incomplete in comparison to the total rate of complications, because in the various papers major complications were not always shown. Therefore, a statistical analysis does not make sense. However, this table shows the wide range of variability of major complications in the different entities. * In the IS group one study on fusion down to the pelvis [69] showed the highest rate of major complications (37%), whilst the range in the IS group, excluding this paper was 0 – 9,5%.

**Table 3:** List of individual complications occurring in the different scoliosis aetiologies as found in the reviewed literature

Complication	NM	AS	IS	CS	Mixed
Death	6,5%	(3S) 2,5%	0,03%		
Pseudarthrosis	(7S) 13,1%	(7S) 17,5%	(5S) 5%		(4S) 23%
Deep wound infection	(3S) 13,2%		(S2) 3,1%		4,7%
Neurologic Complications		(2S) 7,5%	(3S) 1,5%	9%	(4S) 2,7%
Delayed Infection			(2S) 2,9%		
Pedicle screw mispl.			(2S) 15,8%		10,5%
Delayed Paraparesis			X		

Individual complications as have been found in the literature reviewed for the rates of complications [13,68,69,71,77,88,135,138-215] as a descriptive analysis for neuromuscular scoliosis (NM), adult scoliosis (AS), idiopathic scoliosis (IS), congenital scoliosis (CS) and scoliosis of mixed aetiology (Mixed). Whenever there was more than one study found describing a certain complication rate, the number of studies (S) has been given and the rates have been averaged. X = described once.

Risks of spinal fusion include those occurring in any major surgery, such as severe blood loss; urinary infections due to catheterization; pancreatitis; and obstructive bowel dysfunction due to immobilisation during and after surgery [25-31]. The frequency of specific complications, including death is unknown. This is due to problems in reporting such as; mandatory reporting, definitions, interpretation of complications and compliance varies [32]. Information is based on voluntary reporting by clinicians. Other risks of scoliosis surgery are summarised below.

#### Death and neurological damage

The incidence of death as a complication of spinal surgery, for otherwise healthy patients is reported to be less than 1% [33]. In one survey, only one child out of 352 patients died of peritonitis [34] and in a group of 447 patients, two deaths occurred due to pulmonary complications [35]. The life expectancy of patients with a complex neuromuscular condition was significantly reduced by spinal surgery [36]. Another study involving adults with a less than 60% vital capacity measure, 20% had died within 1 year post surgery [37]. In a survey further highlighting these complications [38], 21% were contributed to be secondary to spinal fusion surgery.

Symptoms of neurological damage post-surgery include; partial or total paraplegia, quadriplegia, or peripheral nerve deficit [25,39]. Neurological deficits can result from vascular, metabolic, or mechanical complications of spine surgery [40-51]. Published cases include migration of bone graft into the spinal canal [48]; breakage of implants [52]; penetration of instrumentation into the spinal canal [49] and compression of the nerve roots by components of implants [39].

#### Loss of normal spinal function

In each spinal surgery case there is an irreversible loss of the normal active range of movement in the spinal column [53-55], including the non-fused segments [56-58]. When compared with control subjects, the ability of surgical patients to side flex was reduced by 20–60% [59]. This loss of spinal mobility has gained little significance in the literature, especially in relation to the detrimental effects upon patient health, function, and quality of life. Winter et al. [59] argued that *'it has long been a clinical observation by surgeons who manage scoliosis that patients seem to function well and be relatively unaware of spinal stiffness, even after many motion segments have been fused.' *No data in support of this observation is provided. In actual fact, it has been shown that in non-surgical cases, pain increases as flexibility is reduced [60].

#### Strain on un-fused vertebrae

The post surgical rigid spine causes strain on the un-fused parts of the skeletal framework [54-59] and in a severe case, a woman sustained stress fractures to the pelvis [61]. More commonly reported are post surgical degenerative changes, which occur in young adults [62] and in adults, sometimes within 2 years post-surgery [52]. A higher degree of correction results in a higher rate of degenerative osteoarthritis and the high stress on the rigid spine means that even low impact can cause serious injuries [63]. Surgeons now recommend that in surgically treated scoliosis patients, '*trauma physicians should have a high index of suspicion for potential spinal injuries above a previous multi-level fusion' *[63].

#### Post-surgery pain

Pain is the primary indication for re-operation [64-66]. The mechanism for increased neck and back pain after surgery is not well understood [67]. Bridwell [10] suggests that late-developing pain could be a complication of surgery, or an effect of aging, or *'perhaps a focus on the disability associated with spinal deformity and surgical treatment.' *But the answer for surgeons seems to be to re-operate [68]. Among 190 patients, 19% required re-operation within 2 to 8 years after surgery [67]. For 27 patients who sought treatment 59% felt their pain had been reduced, but 41% did not feel a reduction in their pain levels, and a further 26% were very unhappy with the outcome [68]. Among 34 patients with significant post surgical pain, 56% reported reduced pain after additional surgery, while 44% did not; in the same study, 2 patients who did not have pain before surgery reported pain in the follow up [69].

Pain at the iliac graft site, first noted in 1979, has now been formally published [70,71]; of 87 patients, 24% complained of pain at the graft site, with 15% reporting severity sufficient to interfere with daily activities. As reported by the authors such problems with iliac crest grafting have been severely neglected in literature, especially problems associated with rib-resection.

#### Infection and inflammatory processes

Infections from surgery may manifest months or years later [72-81] and has been detected more than 8 years after surgery, with 5 to10% of patients developing deep infections at 11–45 months after surgery [77,78] and in some cases, leaving the spinal cord exposed to injury [78]. Infections reportedly are becoming more common, perhaps due to larger instrumentation used [77] or perhaps due to the increasing prevalence of multi-drug resistant bacteria in hospital settings. Inflammatory responses to metallic instrumentation can occur independently or in conjunction with infections [79]. Particulate debris from implants can stimulate an autoimmune response that can result in bone deterioration [80]. In most cases, additional surgery to remove instrumentation and to treat the wound is required [81].

Infection may also be transmitted through blood transfusions needed to replace the large amounts of blood lost during invasive procedures [82] and a similar risk occurs with the use of allograft [83]. Some have reported to be infected with HIV following this type of surgery [84]. In a survey of spine surgeons, 41% of those using allograft reported having concerns about the risk of disease transmission and 88% of those make it a policy to inform patients or parents [85].

#### Curvature progression

Some curvatures continue to progress after spinal fusion due to broken rods or other failure of instrumentation. Renshaw [13] has said that, *"One would expect that if the patient lives long enough, rod breakage will be a virtual certainty." *Furthermore, discomfort may occur when any pressure is placed against the back; this is especially problematical with newer bulky instrumentation implanted in thin patients [10].

'Pseudarthrosis' ('false fusion') or failure of the bone graft, which constitutes the spinal fusion, can occur years after surgery and can be difficult to diagnose [86,87]. Among 74 patients treated surgically between 1961 and 1976, pseudarthrosis occurred in 27% of patients within a few years of surgery [88]. For adult patients, 15% had failure of fusion and/or instrumentation requiring additional surgery [89]. Curvatures may continue to progress in young children despite a rigid fusion, due to a *'crankshaft phenomenon' *in which spinal growth causes rotation around the fusion [90,91].

#### Decompensation and increased sagittal deformity

**Figure 3:**
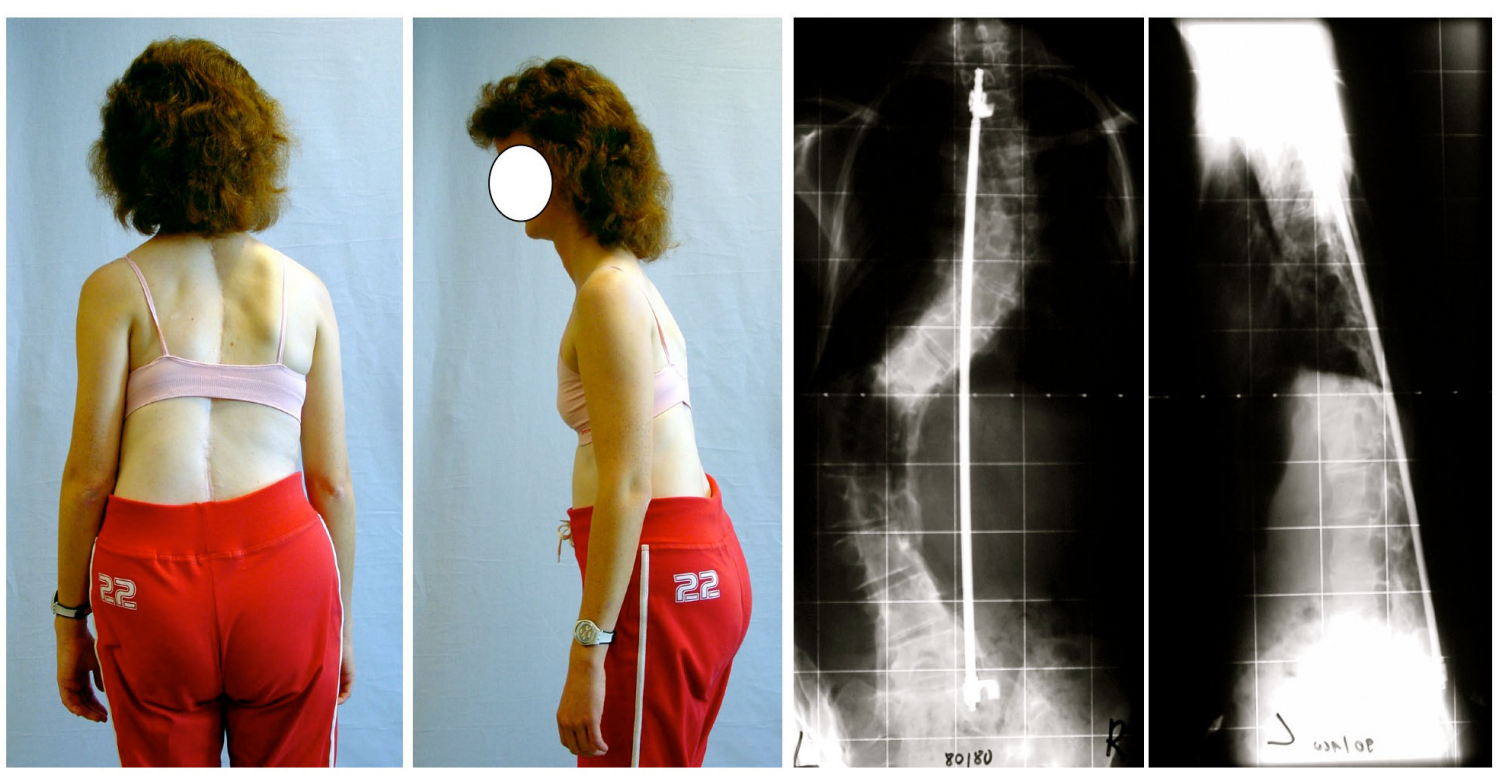
**Ventral decompensation after spinal fusion**. After operation this patient was unable to walk upright. The implant fixed the patient in forward bent position.

**Figure 4:**
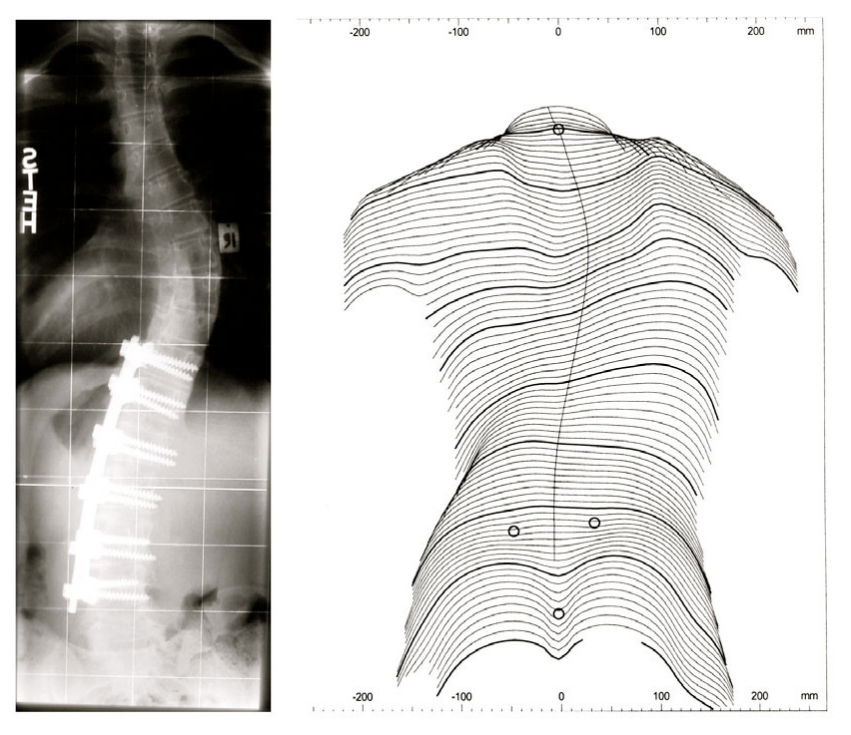
**Lateral decompensation after fusion of the lumbar curve**. The lumbar curve has been fused; the thoracic curve progressed leading to a decompensation to the thoracic convex side. Because of the imbalanced appearance the patient was dissatisfied. There was no cosmetic/psychological benefit in this case and therefore this surgery should perhaps not have been performed.

Beginning with Harrington rods, surgeons have experimented with instrumentation of increasing complexity and bulk to hold spinal fusions in place [24]. Each new variety of instrumentation has brought with it new problems [24]. One of the ongoing problems has been decompensation or the development of new deformities involving changes in sagittal contours and coronal balance of the body as a result of surgery [92-97] (Figure 3 and 4). Reducing the lateral curvature in thoracic scoliosis can exacerbate the sagittal deformity and cause flattening of the cervical, thoracic and/or lumbar spine beyond that which caused the deformity itself [94-97]. Development of *'flatback' *is a painful condition with potentially devastating complications such as disability [98]. In response to such discoveries, focus is shifting towards the sagittal contours and coronal balance of the spine [10].

#### Increased torso deformity

Despite the application of force to straighten and de-rotate the spine during surgery, the rib hump can worsen after surgery [99-102]. Even when rib hump magnitude improves postoperatively, much of the correction can be lost and in many patients the situation is eventually worse than before surgery. In response, surgeons increasingly use costoplasty to assure an improved appearance, by excising the ribs that comprise the prominence [103]. This procedure can in actual fact cause a progressive scoliosis [9] and the destabilising effects of rib removal can also result in a disabling condition called *'flail chest' *in which the normal function of the rib cage is permanently compromised [104]. Rib resection excises a substantial part of the functional components of the chest but the effects on chest expansion has not been documented [24]. However, this procedure has been shown to reduce the volume of the chest cage and to substantially impair pulmonary function [24].

#### Other long-term complications

The complexity of spinal surgery is reflected in the diversity of complications that may occur months or years later. Given the time delay and difficulty in diagnosis, it is likely that only a minority of such events are recognised as surgical complications and when investigated are then recognised as being related to the surgery [88,105-114] (Table 4.).

**Table 4:** List of other long-term complications as found in literature [24]

Complication
Curvature progression after surgery [10,13,86-91]
Decompensation [92-97]
Increased sagittal deformity [98]
Increased torso deformity [99-104]
Emotional breakdown [88]
Gastrointestinal bleeding (late complication 6 years post surgery) [105]
Subarachnoid-pleural fistula [106]
Blindness due to central retinal artery occlusion [107]
Kidney failure due to compression of a ureter [108]
Nerve root injury and degeneration due to compression [109]
Recurrent meningitis [110]
Chronic intermittent vomiting [111,112]
Cast syndrome [113,114]

#### Salvage surgery

Due to such complications outlined above more re-operation is necessary, sometimes referred to as 'reconstructive,' 're-corrective,' 'revision,' or 'salvage' surgery [115]. Even stable fusions may fail in response to sudden force, for example, in the event of automobile accidents [116,117]. Some authors suggest that patients and their parents should be advised that it may take more than one operation [24]. Documented cases of patients having had 5 or more salvage surgeries [69], as in one study, 22% of patients needed a total of 28 additional operations and of 110 adolescent patients 21% required implant removal [118]. Complication rates vary; failure of fusion has been found in more than 50% of treated patients [24] and among 25 adult patients, 40% required salvage surgery [119]. Even when a solid fusion has been obtained by the time of re-operation, removal of instrumentation *'may lead to spinal collapse and further surgery' *[120].

From the patient's perspective, the preferred plan of action would likely to be based upon avoiding unnecessary risk i.e. avoiding surgery, or to keep it as the final option, once all conservative measures have failed. Under this premise, every effort should be undertaken to improve non-operative treatments for at least adolescent idiopathic scoliosis (AIS), the most common form of scoliosis which is regarded to be relatively benign [121]. Other forms of scoliosis may have worse prognoses [4], however real long-term natural history studies do not exist for every single possible form of scoliosis.

Recently claims have been made for a randomised controlled trial (RCT) on brace treatment [122-124], although there is some evidence, that conservative treatment approaches can influence natural history of the disease and decrease the rate of progression [7].

Prospective controlled studies on in-patient rehabilitation and bracing [125-127] and consistent results in retrospective studies [7] justify the recommendation of at least grade B research [128]. Therefore, to perform a RCT on bracing and withhold treatment on half of the patient population with significant curves until surgery may be recommended would be unethical [129]. In view of the fact that there is no evidence that health related signs and symptoms of a scoliosis can be changed by spinal fusion in the long-term [24,130-134], a clear medical indication cannot be derived from most scoliosis conditions [129,131-134]. In the light of an actual publication on adolescent idiopathic scoliosis with a prospective design [135], showing the short-term risks of scoliosis surgery to be more than 3 times higher than previously expected from retrospective reviews, the matter of surgical indications at present should be investigated more closely in order to improve the patient's safety.

The paper by Martha Hawes [24] contains very comprehensive accounts of the reported complications of scoliosis surgery until early 2006. Just recently, new papers on this topic appeared [130,135] and in the light of recent discussions about the specific indications for scoliosis surgery [130-132], a review on this topic seems desirable. The knowledge of the rate of complications of scoliosis surgery may enable us to establish a cost/benefit relationship for this intervention and to improve the quality of the advice given to prospective patients. This study will also address the question as to whether the risks of surgery are small enough to justify the *'wait and see – observation only' *strategy, which is widely accepted [5].

The purpose of this review therefore, is to present the research available on the rate of complications in scoliosis surgery.

## Methods

Exclusion and inclusion criteria for the selection of studies in this review

### Types of studies included

all types of studies, retrospective and prospective ones, reporting on the rate of complications related to scoliosis surgery have been included.

### Types of participants included

patients with any type of scoliosis

### Types of participants excluded

with complications not due to scoliosis surgery.

### Type of intervention

surgery.

### Search strategy for identification of the studies

Pub Med and the SOSORT scoliosis library [136,137], limited to English language and bibliographies of all reviewed articles.

The search strategy included the terms; 'scoliosis'; 'rate of complications'; 'spine surgery'; 'scoliosis surgery'; 'spondylodesis'; 'spinal instrumentation' and 'spinal fusion'.

### Study selection

An electronic search was performed and the studies were selected based on title, abstract and key words. When appropriate, full copy of the articles were printed in order to determine whether or not they met with the inclusion criteria. Additionally, the references of all included articles were checked for further papers that might meet the inclusion criteria. If two papers were found analysing the same group of patients, the most recent paper or the one with the largest sample of patients was selected for inclusion.

## Results

The search carried out on 1^st ^of February 2008, with the key words "scoliosis", "surgery", "complications" revealed 2590 titles, which not necessarily attributed to our quest for the term "rate of complications". 287 titles have been found when the term "rate of complications" was used as a key word.

Of these, 23 papers were found to report on the rate of complication in patients with neuromuscular scoliosis [138-160], 11 papers were found to report on the rate of complication in patients with adult scoliosis [68,161-170], 13 papers were found to report on the rate of complication in patients with idiopathic scoliosis (11 with a general rate of complications) [13,69,135,171-181] and 4 papers were found to report on the rate of complication in patients with congenital scoliosis [182-185]. One paper included patients with early onset scoliosis [186] and one included patients with congenital heart disease [187]. 11 papers reported on the rate of complication in patients with scoliosis of mixed aetiologies [88,188-197] and one in patients after re-operation [198].

Three papers were found to report on problems associated with pedicle screw fixation [199-201], 5 reported on problems associated with thoracoscopic procedures [202-206] and one on problems associated with vertebral body stapling [207].

There were also papers reporting on the rate of certain complications, like pseudarthrosis [208-213], retrolisthesis [214], delayed paraparesis [215], delayed infection [77] and problems with posterior iliac crest bone crafting [71].

In 17 studies, the main focus was on complications, whilst in the others complications have been reported additionally to results, unfortunately most of these utilised different definitions, some of them focussing specifically upon certain complications. Within some of these studies, differences have been made between minor and major complications, however, in most of the articles, the borderline between major and minor have been drawn more or less at random. In one paper a clear definition is given [167], but this definition unfortunately, is not used as a valid general standard.

Major complications were considered to be deep wound infection, pseudarthrosis, transition syndrome, neurologic deficit, and death. Minor complications considered were asymptomatic instrumentation failure (without loss of correction), instrumentation prominence requiring removal, and proximal or distal *'junctional segmental kyphosis' *(5–10 degrees) or subsequent disc space narrowing of 2–5 mm without clinical symptoms.

The pooled overall rate of complications for the different aetiologies can be seen on table 1. The pooled rate of major complications is listed on table 2 and the list of complications found within the reviewed papers can be seen on table 3 and 4.

## Discussion

The prevalence of complications in scoliosis surgery seems quite high. The variation of the rates surely is dependent on the surgical procedure performed, on the specification of the subset of patients investigated in the various studies and on the distribution of Cobb angles in the different samples of patients. For instance, there are many subsets of conditions, which have to be regarded as neuromuscular (Duchenne muscular dystrophy, myelomeningocele patients, cerebral palsy, neuropathic scoliosis, spinal muscular atrophy and poliomyelitic scoliosis). The adult population may consist of adult patients with idiopathic scoliosis or of patients with degenerative deformity. Even the subset of patients with idiopathic scoliosis may contain more or less patients with early onset scoliosis, patients with different Cobb degrees and different maturity. Congenital scoliosis is no uniform condition as well. Therefore, a standardization of patient subsets does not seem possible.

The definition of major and minor complications also varies in these studies. Some authors report the major complications and some report the whole rate of general complications (Table 1 and 2.).

In one study, a re-operation due to instrumentation prominence [167] has been defined as a minor complication as were *'junctional kyphosis' *and disc space narrowing. However, these complications may lead to a re-operation decades after surgery and then might cause major problems. Also, the high rate of pedicle screw misplacements [199-201], thought to be asymptomatic after operation might in fact cause problems in future years after surgery, as has been found in other complications [24].

The follow-up time is also different in the different studies. Long-term follow-up studies have not been found.

As highlighted by Hawes [24], the complexity of spinal surgery is reflected in the diversity of complications that may result months or years later. Given the time delay and difficulty in diagnosis, it is likely that only a minority of such events are recognized as surgical complications.

The data on the rate of complications seems rather in-homogenous and incomplete. Unfortunately there is no mandatory reporting of complications, neither is there a standardized way.

However, the high variability of complication rates could be clarified should a mandatory reporting system exist, reporting on all types of spinal implants in a standardized way. With a spreadsheet list of all recognized complications [24] a 2-year, 5-year, 10-year and 20-year rate of complications could be established for all implants available including the complications 'progression after operation', 'Increased torso deformity' and 'coronal and/or sagittal decompensation after surgery' which are often not really registered by the surgeon [24,86-104,130].

There is a relatively high rate of complications in patients with neuromuscular scoliosis and the benefit for the patient remains questionable as outlined below:

*"Cardio-respiratory function and life expectancy are not improved, but most patients and families are very satisfied by the comfort brought about by the surgical operation*"[159].

*"Surgery should be considered as soon as frontal or sagittal collapse of the spine is observed. However surgery does not result in respiratory improvement, or extending life expectancy" [*160].

The signs and symptoms of AIS obviously are not significantly influenced by surgery [24]. Patients with rare diseases associated with scoliosis like Prader Willi syndrome do not clearly benefit from spinal fusion [133] and surgery of patients with congenital scoliosis has also specifically been under criticism recently [134]. So called long-term studies reporting on congenital scoliosis patients reveal follow-up periods of 3–6 years with most of the patients being still before the pubertal growth spurt at final follow-up [216-219].

To conclude from single case reports that; *"the early fusion prevented the customary severe progression of this condition and early death due to cor pulmonale" *[220,221], seems biased when there could be the possibility that even without surgery cor pulmonale would not necessarily be the consequence of an untreated congenital scoliosis. The question raised by this research is; where is the research and long-term case reports where patients actually did experience severe problems due to surgery? Without such research one can only assume that the rates of complication may be even higher than those reported [222].

What specific evidence is there to support scoliosis surgery? The signs and symptoms of any kind of scoliosis obviously cannot be changed by scoliosis surgery and long-term beneficial effects have not been reported yet as there are no studies presenting long-term risks [24].

No report of the long-term surgical outcome (balance, rate of fusion, rib hump correction, cosmetic correction, pain, and patient satisfaction) is available for any series. Further prospective studies including these parameters will be required to determine the real benefit of such procedures to the patient [24].

As early as 1973, Paul Harrington envisioned in the future a common database or registry of all SRS members' patient's treatment results [8]. Unfortunately the SRS failed to follow this vision until recently. Instead of achieving long-term evidence for surgical treatment on a higher level and addressing the problems after surgery to attempt to improve patient's safety, the surgical community is presenting large numbers of papers describing HRQL after surgery and related research [223-241]. The problem with such studies however, is that they lack validity as they do not investigate the actual signs of scoliosis or the symptoms of the patient post surgery [242].

Those studies containing psychological questionnaires may be compromised by the dissonance effect [242-246], which applies to all situations that include important decisions to be made. Cognitive dissonance occurs most often in situations where an individual must choose between two incompatible beliefs or actions and there is a tendency for individuals to seek consistency among their cognitions. Unable to face an inconsistency, such as being dissatisfied with a surgical procedure, a person will often change his attitude or action.

Surgery is impossible to reverse, but subjective beliefs and public attitude can be altered more easily. The clinical significance of this is that a patient not satisfied with a surgical treatment may not necessarily publicly admit this, as Moses et al. have described in their paper [242]:

*"Slim objective favourable outcomes correlate with high post-surgical patient satisfaction, while a considerable share of patients with whom a highly favourable outcome has been attained express relatively low post-surgical patient satisfaction. This paradoxical trend may be well understood when applying Cognitive Dissonance Theory. The whole pattern of results point again at highly complex and powerful psychological processes, some of them seemingly irrational"*.

**Figure 5:**
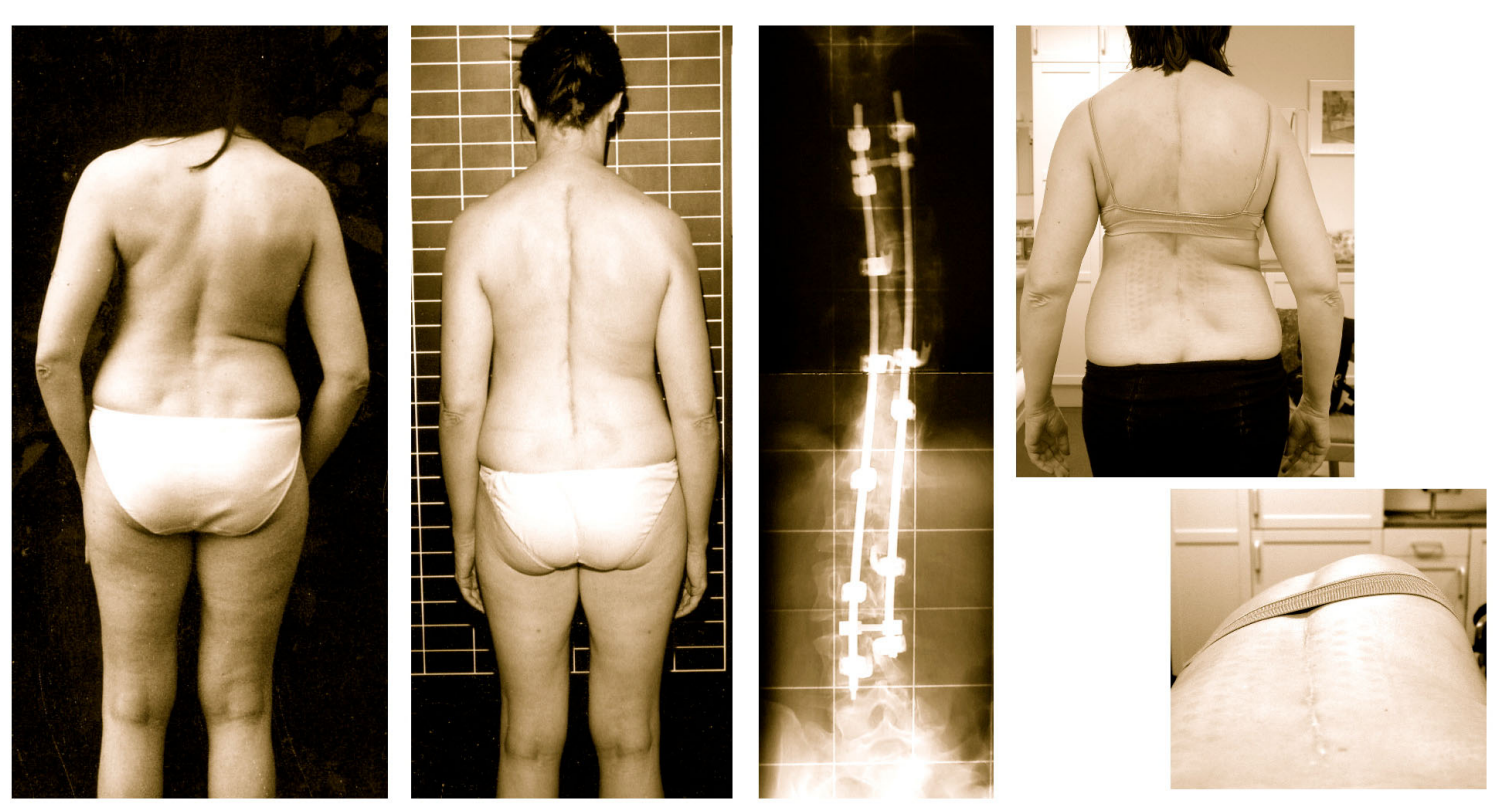
**Balanced appearance with marked rib-hump after surgery**. Although a marked rib-hump is clearly visible after surgery the patient is satisfied with the operation. The rib-hump reappeared after 5 years, however compensation has been maintained. The best cosmetic result was achieved directly after surgery.

The dissonance effect is reflected in the scoliosis literature as well:

*"Patient satisfaction is subjective. It does not reflect the benefits of surgery with respect to the future preservation of pulmonary function in thoracic curves nor the prevention of osteoarthritis in lumbar curves" *[247]. (Figure 5)

**Figure 6:**
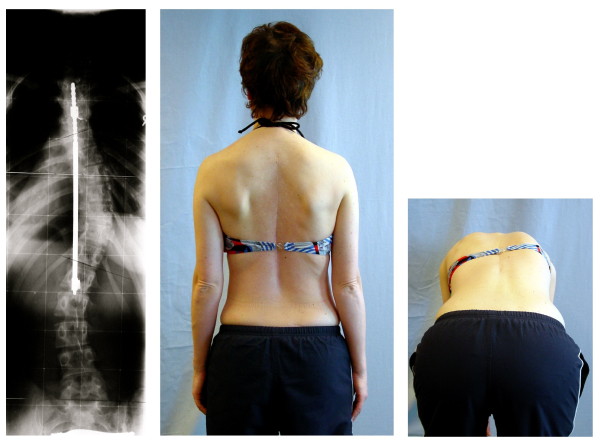
**Excellent clinical result without patient satisfaction**. Excellent clinical result 20 years after Harrington instrumentation. The patient is without pain, however suffers from lack of spinal function although the lumbar spine remained unfused. Additionally, the patient, operated on at the age of 13 years, complained that her parents made the decision. After operation the patient experienced significant functional problems she feels unable to cope with.

**Figure 7:**
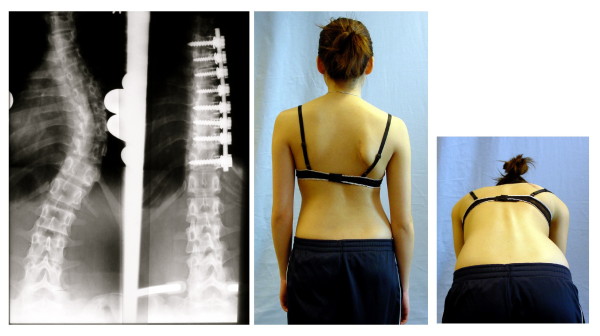
**Excellent radiological result without patient satisfaction**. An *'excellent radiological result' *one year after ventral fusion. But the patient complained about the decompensation (clinical overcorrection) and the visual prominence of the shoulder blade.

**Figure 8:**
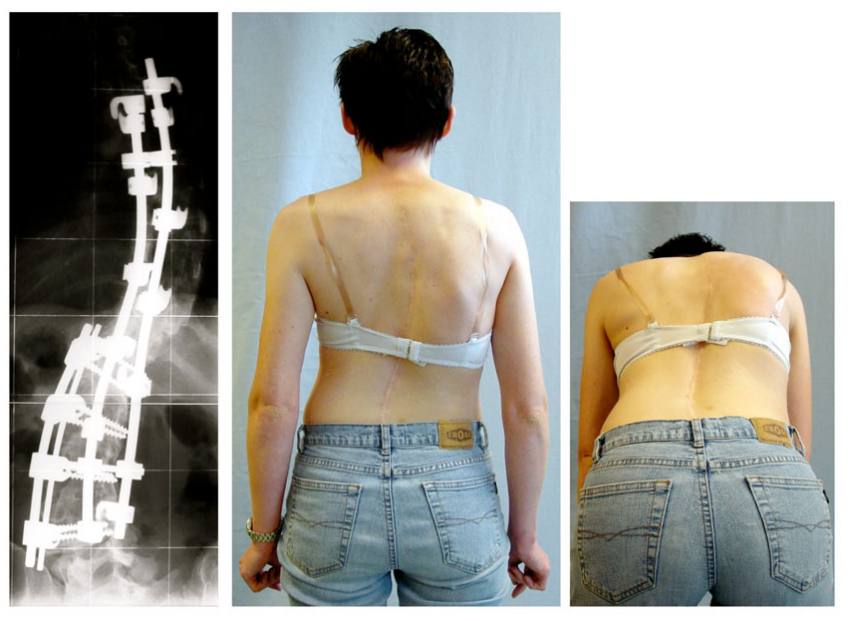
**Not the best clinical result with patient satisfaction**. This patient was satisfied although two operations have been necessary and the rib-hump and decompensation are still visible. This satisfaction may be the result of the dissonance effect [242].

*"Radiographic and physical measures of deformity do not correlate well with patients' and parents' perceptions of appearance. Patients and parents do not strongly agree on the cosmetic outcome of AIS surgery*" [248]. (Figure 6, 7 and 8)

From searching all of the studies based on questionnaires within this review, no evidence can be derived that supports the assumption that patients have experienced benefits from undergoing surgery, as none were able to rule out the cognitive effect of dissonance. Without being able to rule out such effects on the post-operative experience these outcomes do not appear to be reliable [4,249,250].

Today, from the patient's perspective, healthcare professionals have more open questions than answers when approaching the subject of spinal surgery in patients with scoliosis. For example; What are the long-term effects in the elderly; how long does the cosmetic effect of an operation last; is there a prospective controlled study clearly showing that scoliosis surgery really prevents progression in the long term; does the untreated patient really feel more impaired when progressing 10 degrees more in 20 years?

In view of the questions raised by research [129,130], the lack of measurable medical benefit [24] and the high amount of short and long-term risks of the surgical procedures applied, the decision to have surgery does not seem to rely on any valid evidence to support it. The informed patient should perhaps make the final decision after being provided with all the objective facts available. As there is also no clear benefit for the operation in patients with neuromuscular scoliosis [159,160], the indication in these cases deserves to be debated and approved by ethical committees.

Claims for a randomized controlled trial on bracing also seem unethical [129]. To allow growing patients to continue without conservative treatment (a control group) until surgical intervention may be recommended, is completely unethical, especially when one considers the problems with surgery, such as; primary risks; a re-surgery rate, which might be higher than 44% in the long-term [4,24,250] and still undetected future complications which might comprise the elderly patients [4].

It is recognised that this review is limited to the Pub Med/Medline and SOSORT databases and that further database searches would deepen the topic further. To find more variations in a bigger number of papers would not lead to other conclusions as to those that have already been drawn. The lack of standardization of the reports on complications does not allow final conclusions on the numbers per se. The procedure of averaging rates (pooling) as performed in another paper [122] will not permit the estimation of the risk for the individual case. Like the quality of bracing [122] the quality of treatment in surgery is hardly defined in the literature available. It is possible that clinics with smaller rates of complications exist, but in the same way it is possible that clinics with higher rates also exist, which simply may not report their rates of complication publicly [32,222].

A first step into the right direction has been made by the Scoliosis Research Society: In the Scoliosis Research Society Morbidity and Mortality Reports (2002 – 2005) [251] 57 of all patients with spinal fusion (0,2%) died mainly due to cardiac causes, 59 of the scoliosis patients (0,8%) had neurological deficits (in patients with dwarfism mainly), infections between 0,9% (Idiopathic Scoliosis) and 3,4% (neuromuscular scoliosis) and an overall complication rate of 8,6% when all scoliosis aetiologies are concerned (SRS 2002 M & M data abstract [251]). Unfortunately in the years 1994 to 2005, there is a varying percentage of SRS members submitting data, ranging from 35 to 70% (SRS 2005 M & M data abstract [251]). Additionally to that not all types of complications are registered like many of those described in the introduction of this paper. The long-term complications, that may develop years after surgery [24] are not listed in the M & M summaries [251], which are not easily accessible to the public.

In the light of the conflict of interest many spine surgeons have because of their affiliation to industry [251-253], the indication for surgery in the case of scoliosis may well be more appropriately assigned to a more specialized role. For example, a conservative scoliosis specialist that can utilise standardized psychological questionnaires [254] after having discussed all possible benefits and complications of surgery with the patient.

## Conclusion

Scoliosis surgery has a varying but high rate of complications. A medical indication for scoliosis surgery cannot be established in view of the lack of evidence found within this review. Long-term risks of scoliosis surgery have not yet been reported. Mandatory reporting for all spinal implants in a standardized way using a spreadsheet list of all recognized complications to reveal a 2-year, 5-year, 10-year and 20-year rate of complications should be established which may help develop a more clear indication for surgery and a more accurate account of the complications of surgery. Trials with untreated control groups in the field of scoliosis are unethical, when the control group is exposed to the risks of undergoing surgery.

## Competing interests

The authors declare that they have no competing interests.

## Authors' contributions

HRW manuscript writing, research of databases and Figures, DG manuscript writing, copyediting and research of databases. All authors read and approved the final manuscript.
